# Syndemic effects of HIV risk behaviours: results from the NHANES study

**DOI:** 10.1017/S095026881900133X

**Published:** 2019-07-12

**Authors:** L. Smith, C. Cao, X. Zong, D. T. McDermott, S. Stefanac, S. Haider, S. E. Jackson, N. Veronese, G. F. López-Sánchez, A. Koyanagi, L. Yang, I. Grabovac

**Affiliations:** 1 Cambridge Centre for Sport and Exercise Sciences, Anglia Ruskin University, Cambridge, UK; 2 Division of Public Health Sciences, Department of Surgery, Washington University School of Medicine, St Louis, MO, USA; 3 Division of Psychology, School of Psychology and Sports Sciences, Anglia Ruskin University, Cambridge, UK; 4 Institute of Outcomes Research, Center for Medical Statistics, Informatics and Intelligent Systems, Medical University of Vienna, Vienna, Austria; 5 Ludwig Boltzmann Cluster Arthritis and Rehabilitation, Vienna, Austria; 6 Department of Social and Preventive Medicine, Center for Public Health, Medical University of Vienna, Vienna, Austria; 7 Department of Behavioural Science and Health, University College London, London, UK; 8 National Research Council, Neuroscience Institute, Aging Branch, Padova, Italy; 9 Faculty of Sport Sciences, University of Murcia, Murcia, Spain; 10 Research and Development Unit, Parc Sanitari Sant Joan de Déu, Fundació Sant Joan de Déu, CIBERSAM, Sant Boi de Llobregat, Barcelona, Spain; 11 Department of Epidemiology, Center for Public Health, Medical University of Vienna, Vienna, Austria

**Keywords:** Adults, HIV, NHANES, risk factors, syndemic theory

## Abstract

The aim of the present study is to use the syndemic framework to investigate the risk of contracting HIV in the US population. Cross-sectional analyses are from The National Health and Nutrition Examination Survey. We extracted and aggregated data on HIV antibody test, socio-demographic characteristics, alcohol use, drug use, depression, sexual behaviours and sexually transmitted diseases from cycle 2009–2010 to 2015–2016. We carried out weighted regression among young adults (20–39 years) and adults (40–59 years) separately. In total, 5230 men and 5794 women aged 20–59 years were included in the present analyses. In total, 0.8% men and 0.2% women were tested HIV-positive. Each increasing HIV risk behaviour was associated with elevated odds of being tested HIV-positive (1.15, 95% CI 1.15–1.15) among young adults and adults (1.61, 95% CI 1.61–1.61). Multi-faceted, community-based interventions are urgently required to reduce the incidence of HIV in the USA.

## Introduction

A total of 39 782 US residents were diagnosed with human immunodeficiency virus (HIV) infection in 2016 [1]. Of those diagnosed, 44% were black/African Americans and 67% were gay and bisexual men [2]. Importantly, those aged between 20 and 39 years seem to be at very high risk of contracting HIV [1]. Additionally, the number of people contracting HIV in 2016 in the USA is likely to be higher than this. The true prevalence rate is evasive, as prevalence rates are difficult to measure directly due to a proportion of people living with HIV that have not been diagnosed and those newly found that have not been reported to local surveillance programmes [3].

As in many industrialised countries, data suggest that the rates of HIV diagnosis in the USA have remained relatively stable in the last 5 years, although inflicting a large number of people [1]. In order to achieve a reduction in incidence, it is important to identify key risk factors associated with HIV contraction in the US population. For HIV transmission, these include various biomedical and social factors which are often cited as: having multiple sexual partners, alcohol use and drug and polydrug use, having a sexually transmitted infection, unprotected sex, depressive symptoms, being a black/African American, gay and bisexual men, and those from a lower socio-economic status [4–9]. However, it is likely that these risk factors cluster and have a synergistic effect that increases the risk of contracting HIV.

*Syndemic theory* may be the theoretical framework best suited to explain the contraction of HIV. A syndemic framework focuses on complex interactions of (multiple) diseases but also social and environmental factors contributing to the excess burden of disease in a population [10, 11]. Therefore, syndemics include epidemics of both the disease and the social conditions that contribute to the proliferation of disease [12]. Several studies have investigated syndemic theory in order to explain HIV risk behaviours in specific contexts with specific populations [13–16]. However, these studies are limited as they have not used HIV infection as an outcome, but rather HIV risk behaviours, which may not necessarily lead to an infection. To our knowledge, no study has to date applied the syndemic framework to explain the contraction of HIV in a national representative sample of the US population.

The aim of the present study is to expand on and add to the existing literature by using the syndemic framework to investigate the risk of contracting HIV in the US population.

## Methods

### Study population

The National Health and Nutrition Examination Survey (NHANES) was designed to evaluate the prevalence of health, nutrition and potential lifestyle risk factors among the civilian non-institutionalised US population up to 85 years old. The design of NHANES has been detailed elsewhere [17]. In brief, NHANES surveys a nationally representative complex, stratified, multistage, probability clustered sample of about 5000 participants each cycle in 15 counties across the USA. The participants were required to attend the physical examination in a mobile examination centre (MEC). The research conformed to the principles embodied in the Declaration of Helsinki of 1975, as revised in 2008. The NHANES obtained approval from the National Center for Health Statistics Research Ethics Review Board and participants provided written informed consent. We extracted and aggregated data on socio-demographic characteristics, HIV antibody test, alcohol use, drug use, depression, sexual behaviours and sexually transmitted diseases (STDs) from cycle 2009–2010 to 2015–2016. We restricted our study sample to men and women aged 20–59 years because of the upper age limit of the HIV test result used in the present analyses.

### HIV-positive

HIV-positive results were accessed by NHANES laboratory procedures. Participant's blood was drawn by trained phlebotomists in the MEC and processed, stored and shipped to the Division of AIDS, STD and TB; National Center for HIV, STD and TB Prevention; National Centers for Disease Control and Prevention. The serum specimens were tested using an enzyme-linked immunosorbent assay (ELISA) to detect the antibody to HIV, followed by confirmatory Western blot for those with positive ELISA tests [18]. The HIV antibody test result includes ‘Positive’, ‘Negative’ and ‘Indeterminate’. We aggregated the results into a binary variable: HIV-positive *vs.* HIV-negative by excluding indeterminate results due to the uncertain confirmation.

### Multiple risk behaviours

#### Drug use

Lifetime drug use was self-reported during the MEC interview. Three metrics on drug use were derived: marijuana or hashish, cocaine/heroin/methamphetamine and illegal drug. Both men and women were asked ‘The following questions ask about the use of drugs not prescribed by a doctor. Have you ever, even once, (1) used marijuana or hashish? (2) used cocaine, crack cocaine, heroin or methamphetamine? (3) used a needle to inject a drug?’ with response options of ‘Yes’, ‘No’, ‘Refused’ and ‘Don't know’. We aggregated the responses into a binary variable: no drug use *vs.* at least one drug use. Further, injecting illegal drug (yes/no) was classified as a binary variable in sensitivity analyses.

#### Depressive symptoms

Depressive symptoms were assessed using the Patient Health Questionnaire (PHQ-9), a valid nine-item depression screener asking about the frequency of symptoms of depression over the past 2 weeks [19]. Each item was scored on a 0–3 scale. The total score of PHQ-9 ranged from 0 to 27. We categorised depressive symptoms by PHQ-9 score as ‘none or minimum’ (0–4), ‘mild’ (5–9), ‘moderate’ (10–14), ‘moderately severe’ (15–19) and ‘severe’ (20–27) for severity. For current analyses, participants who scored 10 or more were combined into one group as clinically relevant depression. Such diagnosis has shown a sensitivity of 88% and a specificity of 88% for major depression [19].

#### Sexual behaviours and STDs

The sexual behaviour questionnaire was completed by participants at the MEC. Information on lifetime and current sexual behaviour and history of STDs was collected. We derived the number of sex partners, condom use and STDs. The number of sexual partners last year was derived for men and women, respectively. We summarised the total number of sex partners (same or opposite sex) in the past year for each participant who reported having (performing or receiving) any kind of sex. Due to the large inter-individual variation in the number of sex partners, we used a dichotomised variable to indicate none or one *vs.* multiple (⩾2) sexual partners in the past year to capture multiple sexual partners. Also, we categorised the condom use into a binary variable: Always (never had sex without condom) *vs.* Not always (occasionally or always had sex without condom) in the past year. Additionally, both men and women were asked whether the doctor ever told them that they had genital herpes, genital warts, gonorrhoea, chlamydia and HPV (only for women), respectively. We summarised STDs as one binary variable: Yes (Doctor ever told had at least one of these STDs) *vs.* No.

Finally, to account for the syndemic effect of multiple HIV risk behaviours, we generated an accumulative score for five behaviours, including drug use, depression symptoms, multiple sex partners, condom use and STDs, into one continual variable.

### Socio-demographic characteristics

Data on age, sex and a range of characteristics were extracted. Based on self-reported race and ethnicity, participants were classified into one of the four racial/ethnic groups: Non-Hispanic White, Non-Hispanic Black, Hispanic, other. Participants' household annual income and education levels were classified into three groups: <$25 000, $25 000–74 999 and ⩾$75 000, and less than high school, high school and above high school, respectively.

### Patient and public involvement

Patients were not involved in the design of the present study.

### Statistical analysis

Survey analysis procedures were used to account for the sample weights, stratification and clustering of the complex sampling design to ensure nationally representative estimates. Descriptive characteristics were analysed separately in men and women due to the documented gender difference in HIV prevalence. We summarised weighted proportions for categorical variables by gender.

Due to the established age difference in HIV prevalence, we carried out the weighted regression among young adults (20–39 years) and adults (40–59 years) separately. Due to rare events of HIV, we corrected our results by using firthlogit program to reduce the bias of logistic regression [20]. Multivariable models were conducted to estimate the associations of each risk behaviour and the accumulative score of risk behaviours with HIV-positive, adjusting for age, race/ethnicity, household income and education in adults 20–39 and 40–59 years, respectively.

Sensitively analyses were carried out by limiting drug use to injection, defined as injecting illegal drug (yes/no). All statistical significance was set at *P* < 0.05. All statistical analyses were performed using STATA version 14.0 [21].

## Results

There were 5230 men and 5794 women aged 20–59 years who had data on HIV antibody test and risk behaviours (Table 1). A total of 0.8% of men and 0.2% of women were tested positive for HIV. The weighted mean of HIV risk behaviour scores was 1.86 (s.e., 0.02) among men and 1.79 (s.e., 0.02) among women. However, the prevalence of moderate-to-severe depressive symptoms was higher among women (11%) than that among men (6.7%). Moreover, 20% of women reported doctors diagnosed STDs in our sample, higher than that in men (6.1%), primarily due to 11% of women reporting having been told by a doctor that they had HPV.

**Table 1.** Characteristics of adults aged 20–59 years from the NHANES (2009–2016), by gender^a^

		Men	Women
	*N*	5230 (100)	5794 (100)
Age group			
20–39	*n* (%)	2626 (48)	2908 (48)
40–59	*n* (%)	2604 (52)	2886 (52)
Race			
Non-Hispanic white	*n* (%)	2500 (71)	2594 (69)
Non-Hispanic black	*n* (%)	1205 (11)	1448 (14)
Hispanic	*n* (%)	1525 (18)	1752 (17)
Education			
<High school	*n* (%)	1220 (16)	1188 (13)
High school	*n* (%)	1340 (24)	1190 (19)
>High school	*n* (%)	2670 (60)	3416 (68)
Annual household income			
<25 000	*n* (%)	1426 (18)	1812 (22)
$25 000–75 000	*n* (%)	2238 (39)	2441 (40)
>75 000	*n* (%)	1572 (43)	1541 (38)
HIV-positive	*n* (%)	52 (0.8)	20 (0.2)
Multiple risk behaviours	Mean (s.e.)	1.86 (0.02)	1.79 (0.02)
Drug use	*n* (%)	3009 (66)	2581 (57)
Marijuana or hashish	*n* (%)	3006 (66)	2577 (57)
Cocaine/heroin/methamphetamine	*n* (%)	1197 (26)	719 (16)
Inject illegal drug	*n* (%)	137 (3.3)	77 (1.6)
Depression	*n* (%)	406 (6.7)	742 (11)
Multiple (⩾2) sex partners	*n* (%)	1482 (25)	1472 (22)
Condom use			
Always	*n* (%)	1032 (23)	987 (20)
Sexually transmitted disease	*n* (%)	276 (6.1)	894 (20)
Genital herpes	*n* (%)	110 (2.3)	299 (6.9)
Genital warts	*n* (%)	148 (3.5)	270 (6.5)
Gonorrhoea	*n* (%)	22 (0.3)	16 (0.3)
Chlamydia	*n* (%)	32 (0.6)	74 (1.2)
HPV	*n* (%)	–	469 (11)

a The weighted proportions for categorical variables were presented.

Table 2 summarises multivariable logistics regression of the association between the cumulative HIV risk behaviour variable and the odds of being tested HIV-positive, among young adults 20–39 years old and adults 40–59 years, respectively (for associations of each risk behaviour and HIV-positive, see Supplementary Table). Each increasing HIV risk behaviour was associated with elevated odds of being tested HIV-positive (1.40, 95% CI 1.40–1.41) among young adults and adults 40–59 years old (2.02, 95% CI 2.02–2.03). In addition, striking disparity patterns among participants being tested HIV-positive were observed according to the socio-demographic factors, consistently in adults of both age groups. For example, men were more likely to be tested HIV-positive than women, although the multivariable-adjusted OR was slightly higher among adults 40–59 years old (5.09, 95% CI 5.07–5.11) compared to young adults 20–39 years old (4.01, 95% CI 3.98–4.03). Ethnical disparities were strong among younger adults; non-Hispanic blacks had 20.4 (95% CI 20.3–20.3) higher odds of being tested HIV-positive compared with non-Hispanic whites. Other racial/ethnical disparities observed included non-Hispanic blacks (3.61, 95% CI 3.59–3.62) compared to non-Hispanic whites aged 40–59 years, and Hispanics 20–39 years (2.14, 95% CI 2.12–2.16) and 40–59 years (1.56, 95% CI 1.55–1.56) compared to non-Hispanic whites. Finally, higher annual household income was associated with lower odds of being tested HIV-positive among all age adults (*P* for trend<0.001). In contrast, graded education level was associated with higher odds of being tested HIV-positive (*P* for trend<0.001). Sensitivity analyses defining drug use by injecting illegal drug (yes/no) showed similar results (Table 2).

**Table 2.** Weighted logistic regression models of HIV-positive among adults 20–59 years from NHANES 2009–2016, adjusted for socio-demographic and lifestyle factors

	Odd ratio (95% CI)		
	All	20–39 years	40–59 years
Multiple risk behaviours^a^	1.85 (1.85–1.86)	1.40 (1.40–1.41)	2.02 (2.02–2.03)
Age	1.06 (1.06–1.06)	1.05 (1.05–1.05)	1.04 (1.04–1.04)
Sex			
Women	1 (Reference)	1 (Reference)	1 (Reference)
Men	4.78 (4.77–4.80)	4.01 (3.98–4.03)	5.09 (5.07–5.11)
Race/ethnicity			
Non-Hispanic white	1 (Reference)	1 (Reference)	1 (Reference)
Non-Hispanic black	5.50 (5.49–5.52)	20.4 (20.3–20.3)	3.61 (3.59–3.62)
Hispanic	1.51 (1.50–1.51)	2.14 (2.12–2.16)	1.56 (1.55–1.56)
Annual household income			
<$25 000	1 (Reference)	1 (Reference)	1 (Reference)
$25 000–<$75 000	0.45 (0.45–0.46)	0.65 (0.65–0.66)	0.39 (0.38–0.39)
⩾$75 000	0.38 (0.38–0.39)	0.74 (0.73–0.74)	0.30 (0.30–0.30)
*P* value for trend	<0.001	<0.001	<0.001
Education			
<High school	1 (Reference)	1 (Reference)	1 (Reference)
High school	1.39 (1.38–1.39)	1.27 (1.26–1.28)	1.45 (1.44–1.45)
>High school	1.99 (1.98–1.99)	1.20 (1.19–1.20)	2.39 (2.38–2.40)
*P* value for trend	<0.001	<0.001	<0.001
Multiple risk behaviours^b^	1.82 (1.81–1.82)	1.28 (1.28–1.29)	2.02 (2.02–2.02)
Age	1.05 (1.05–1.05)	1.05 (1.05–1.05)	1.03 (1.03–1.03)
Sex			
Women	1 (Reference)	1 (Reference)	1 (Reference)
Men	5.26 (5.24–5.28)	4.15 (4.12–4.17)	5.70 (5.68–5.72)
Race/ethnicity			
Non-Hispanic white	1 (Reference)	1 (Reference)	1 (Reference)
Non-Hispanic black	5.37 (5.36–5.39)	20.7 (20.6–20.9)	3.45 (3.44–3.46)
Hispanic	1.34 (1.33–1.34)	2.02 (2.00–2.04)	1.30 (1.29–1.31)
Annual household income			
<$25 000	1 (Reference)	1 (Reference)	1 (Reference)
$25 000–<$75 000	0.47 (0.47–0.48)	0.66 (0.65–0.66)	0.42 (0.41–0.42)
⩾$75 000	0.42 (0.42–0.42)	0.75 (0.74–0.75)	0.34 (0.34–0.34)
*P* value for trend	<0.001	<0.001	<0.001
Education			
<High school	1 (Reference)	1 (Reference)	1 (Reference)
High school	1.56 (1.56–1.57)	1.26 (1.25–1.27)	1.71 (1.70–1.72)
>High school	2.29 (2.28–2.30)	1.19 (1.18–1.19)	2.94 (2.92–2.95)
*P* value for trend	<0.001	<0.001	<0.001

a Multiple risk behaviours included drug use, depression, multiple (⩾2) sex partners, never use condom and sexually transmitted disease.

b Multiple risk behaviours included injecting illegal drug, depression, multiple (⩾2) sex partners, never use condom and sexually transmitted disease.

## Discussion

The present study in a representative sample of the US adult population found that men were at a much higher risk of contracting HIV than women, and that Hispanics and non-Hispanic blacks were at a much higher risk than non-Hispanic whites, as well as those who had a higher education (high school education or greater) compared to those with a low level of education. Furthermore, those with a high annual income were at a lower risk of contracting HIV compared to those with a low annual income. Importantly, the present study found that for each additional risk factor one is exposed to, there is an increased risk in the contraction of HIV in young and mid-adulthood.

The present findings support the previous reports that have shown in the US population that men and non-whites are at a greater risk of contracting HIV [1]. This may be owning to a variety of reasons that are related to disparity in health care access as well as a higher prevalence of other STDs but also due to concurrent partnerships which are associated with more risky behaviours [22–25]. However, interestingly our findings both confirm and contradict research in relation to socio-economic status. Previous literature suggests that in the USA those who are from a low socio-economic status are at a greater risk of contracting HIV when compared to those from a high socio-economic status [4]. Indeed, in the present study, we found that those with a high annual income were at a lower risk compared to those from a low, but those with high school level of education or higher were at a greater risk than those without a high school level education. It is indeed possible that the level of education is not a strong predictor of socio-economic status, when compared to annual income, in the USA particularly for those in middle adulthood [26]. Moreover, the relationship between obtained education levels and health outcomes may not be as straightforward [27], with health literacy (i.e. the degree to which patients understand the given information about their health and the ability to make decisions based on these information) providing a possible pathway between the two [28]. However, research has shown that substantial proportions of highly educated people still have low health literacy showing that higher obtained education levels do not equate to higher health literacy [29, 30].

Perhaps most importantly for the first time in a large representative sample of US adults, the present study found that with each additional risk factor one is exposed to the risk of HIV contraction significantly increases in young adulthood and middle adulthood. These findings support the use of a syndemic framework to study and understand HIV contraction risk. These findings suggest that those exposed to or partaking in multiple risk behaviours require urgent multifaceted tailored interventions targeting all risk factors to prevent HIV contraction. Indeed, the removal of just one risk factor can significantly lower the risk of contracting HIV. The greater risk of contracting HIV with an increasing number of risk factors in mid-adulthood compared to young adulthood should be noted. It is likely that those who are older may have been exposed to risk factors for a longer period of time (e.g. used drugs and had unprotected sex for a greater period of time) and thus have a greater opportunity to contract HIV.

The result of the study provides an evidence base for future public health interventions. The classic top-down paternalistic approach that is the most common approach in public health interventions usually not taking into consideration the larger social, economic and behavioural complexities provides short lived effects [31, 32]. These implementation models should give way to more collaborative, community-based and evidence-informed bottom up interventions where several risk factors can be simultaneously addressed within the community [31]. These are even more important when it comes to improving resilience in communities that face great health and social disparity [33].

Strengths of this study include a large representative sample of US adults and the use of syndemic theory to understand HIV contraction and not behaviour. Limitations include a relatively low number of participants with HIV infection and a cross-sectional study design. Moreover, the present study asked about the frequency of symptoms of depression over the past 2 weeks, it is therefore unclear if depression promoted risk behaviours or an HIV diagnosis promoted depression. Finally, literature has suggested that men who have sex with men are at a high risk of contracting HIV. However, such data were not available and thus not included in the present analyses. Future research using a syndemic framework to understand HIV infection should consider incorporating sexual orientation. Similarly, as there has been a reported rise in primary and secondary syphilis infections in the USA [34], which is a known risk for HIV infection, further studies should incorporate a more comprehensive list of STDs. Unfortunately, this variable was also not available in the dataset.

In conclusion, the present study has shown for the first time in a large representative sample of US adults that with increasing exposure to potential HIV risk factors, the risk of HIV contraction significantly increases. Multi-faceted, community-based interventions are urgently required to reduce the incidence of HIV in the USA.

## Supplementary material

For supplementary material accompanying this paper visit https://doi.org/10.1017/S095026881900133X. click here to view supplementary material
