# Supplementary material for: Syndemic effects of HIV risk behaviours: results from the NHANES study
Source: Epidemiol Infect. 2019 Jul 12;147:e241. doi: 10.1017/S095026881900133X (PMC6635805; doi:10.1017/S095026881900133X)
Supplement: Supplementary file 1 [file S095026881900133Xsup001.docx]

| **Supplemental table. Association Between Risk Behaviors and HIV-Positive Among Adults from NHANES 2009-2016, Adjusted for Socio-demographic and Lifestyle Factors^a^** | | | |
| --- | --- | --- | --- |
|  | **Odds Ratio** | |  |
|  | **HIV positive** | |  |
|  | All | **20-39 years** | **40-59 years** |
| Drug use | 1.55 (1.54 to 1.55) | 1.26 (1.25 to 1.27) | 1.72 (1.71 to 1.73) |
| Depression | 1.78 (1.77 to 1.79) | 0.76 (0.76 to 0.77) | 2.14 (2.13 to 2.15) |
| Multiple (≥2) sex partners | 2.68 (2.67 to 2.68) | 4.07 (4.05 to 4.09) | 2.27 (2.26 to 2.28) |
| Never use condom | 0.95 (0.95 to 0.96) | 0.36 (0.35 to 0.36) | 1.63 (1.62 to 1.64) |
| Sexually transmitted disease | 3.52 (3.51 to 3.53) | 1.72 (1.71 to 1.73) | 4.43 (4.42 to 4.45) |
| a For each risk behavior, the logistic regression model was adjusted for age, race, household income, and education level. | | | |
